# Associated Effects of Meaning in Life and Social Adjustment Among Chinese Undergraduate Students With Left-Behind Experiences in the Post-epidemic Period

**DOI:** 10.3389/fpsyt.2021.771082

**Published:** 2021-12-03

**Authors:** Ying Ge, Mei-Qiong Shuai, Jie Luo, Jay L. Wenger, Cun-Yang Lu

**Affiliations:** ^1^Key Laboratory of Emotion and Emotion and Mental Health in Chongqing, Chongqing University of Arts and Sciences, Chongqing, China; ^2^School of Humanities & Social Sciences, Fuzhou University, Fuzhou, China; ^3^School of Psychology, Guizhou Normal University, Guiyang, China; ^4^Social Sciences Division, HACC, Central Pennsylvania's Community College, Lancaster, PA, United States

**Keywords:** post-epidemic period, Chinese undergraduate students with left-behind experiences, meaning in life, social adjustment, dominance analysis

## Abstract

**Background:** The outbreak of COVID-19 has brought about radical changes in social life. The study focuses on a special group, Chinese undergraduate students with left-behind experiences. Specifically, the study addresses how such students feel and grasp the meaning in life and how they adapt to the current social environment after experiencing the impermanence of life. The correlation between the meaning in life and social adjustment in the post-epidemic period is evaluated.

**Methods:** The Meaning in Life Scale and the Social Adjustment Scale were used to test 988 undergraduate students. Multi-factor analysis, correlation, regression, and dominance analysis were performed on the test results.

**Results:** (1) During the epidemic, Chinese undergraduate students generally had low meaning in life scores, including below-average values for life goals, and middle-range scores for social adjustment. (2) Having or not having left-behind experiences had an important influence on the meaning in life and social adjustment of undergraduates: undergraduates with left-behind experiences performed better than those without left-behind experiences in terms of meaning in life, while their social adjustment was weaker than those without left-behind experiences. (3) The zest for life and freedom of life of undergraduates in both groups negatively predicted social adjustment, and zest for life preferentially influenced social adjustment. Zest for life also had a significant effect on life value in the group without left-behind experiences. Zest for life was a priority factor influencing social adjustment.

**Conclusion:** The epidemic and left-behind experiences are important factors influencing the relationship between meaning in life and social adjustment among Chinese undergraduate students.

## Introduction

In late 2019 and early 2020, a “storm” of COVID-19 swept the whole world. Most Chinese were stuck at home, using their devices and screens to communicate and maintain social contact. Screens were now used to convey good news that relatives and friends were safe and bad news about illnesses and death, causing an inordinate amount of tension and stress. Currently, China has entered the phase of regular epidemic prevention and control. Its college undergraduates, with a large portion receiving cross-regional education, rely more on the Internet for distance learning. These students often have to wear masks, show health codes, get vaccinated and undergo nucleic acid tests. Changes in their learning/living environments tended to increase anxiety, depression, confusion, and helplessness (1, 2). In such a context, how should Chinese undergraduate students with left-behind experiences feel and grasp meaning in life? What are the special manifestations and patterns of social adjustment? How is meaning in life related to social adjustment? These questions have important implications for psychological well-being, and they inspire the current research.

Left-behind experience is a concept that is used to describe what a Chinese undergraduate student experiences when they are currently enrolled at a college or University and have lived apart from their out-working parent(s) for more than half a year in the juvenile period (3). Left-behind children represent a particular phenomenon in China's economic development. They account for about 14-26% of the population at institutions of higher education, and as high as 78.24% at vocational colleges (4, 5). Left-behind experiences can generate loneliness during a critical time in a person's development. As a result, Chinese undergraduates with left-behind experiences have underperformed both socially and mentally during the COVID epidemic (6).

Life meaning is a concept proposed by the famous psychologist Viktor Frankl. It includes a person's awareness and pursuit of purposes and goals in life (7). Undergraduate students are in the early stage of youth, a crucial period for personality development and ego identity, and they have the will to actively pursue meaning in life (8, 9). People who have found their meaning in life are psychologically healthier and adapt better socially (10–12). In comparison, those who lack such meaning tend to experience loneliness (13, 14), anxiety, and depression (15). Furthermore, Chinese undergraduate students with left-behind experiences have less momentum in their search for meaning, and the momentum decreases with time (16).

Social adaptation is a process of positive interaction between the individual and the external environment – an environment from which the individual continuously obtains information and makes adjustments (17). Late puberty is a complex time in a person's life. It is a time of physical and mental changes, along with social transitions (18). One study found that parental roles are usually absent in left-behind adolescents' socialization, resulting in lower adaptability (19). However, this may also be a positive factor because it encourages young people with left-behind experiences to deal with problems and enhance their own social adaptability (3).

At present, there are some studies with undergraduates that suggest meaning in life is significantly and positively correlated with social adjustment and serves as an effective predictor of social adjustment (20–22). However, concerning the relationship between meaning in life and social adjustment, there are no direct studies that involve undergraduate students with left-behind experiences. In sum, we believe it is important to address the issues and difficulties that Chinese undergraduate students with left-behind experiences face during this challenging time. Hopefully, interventions can be found and applied.

To this end, the hypothesis of this study is that life meaning and social adjustment of Chinese undergraduates with left-behind experiences have unique characteristics in the post-epidemic period. Meaning of life is correlated with social adjustment, and there are dominant factors. The epidemic and left-behind experiences are important factors influencing the relationship between meaning in life and social adjustment among Chinese undergraduate students.

## Methods

### Participants

The selection criteria for undergraduate students with left-behind experiences include: (1) being currently enrolled at a college or University and once lived apart from their out-working parent(s) for more than half a year in the juvenile period; (2) having an age between 18 and 22, considering gender, grade, residence and number of children; (3) not having a severe physical illness or mental illness. The inclusion criteria for ordinary college students are the same as above (except Item 1). Each participant voluntarily answered all questions and signed an informed consent.

Through convenience sampling method, 1,050 questionnaires were distributed online, and 988 valid ones were recovered, with an effective recovery rate of 94.09%. The distribution of participants is shown in Table 1.

**Table 1:** Distribution of participants.

**Variables**	**Levels**	**Number**	**Proportion**
Gender	Male	463	46.86%
	Female	525	53.14%
Grade	Freshman	343	34.72%
	Sophomore	399	40.38%
	Junior	182	18.42%
	Senior	64	6.48%
Left-behind Experience	Having	421	42.61%
	Not Having	567	57.39%
Only-Child	Yes	436	44.13%
	No	552	55.87%
Origin	Urban	467	47.27%
	Rural	521	52.73%

### Research Tools

#### Meaning of Life Scale (MLS)

The Meaning of Life Scale in this study was revised by Dong (23) with reference to Song (24). It consists of 18 questions in 4 dimensions of life enthusiasm, life goal, life value, and life freedom. Answers were given on a 7-point Likert scale – the higher the score, the greater the sense of meaning in life. The coefficient alpha (α) of the full scale in this study is 0.886.

#### Social Adaptation Scale (SAS)

The Chinese College Student Adjustment Scale (25) consists of 60 questions in 7 dimensions of satisfaction, emotional adaptation, study adaptation, occupational adaptation, self-adaptation, interpersonal adaptation, and campus adaptation. Answers were given on a 5-point Likert scale – the higher the score, the better the social adaptability. The coefficient alpha (α) of the full scale in this study is 0.933.

### Testing Method and Process

We used a MANOVA, a correlation, and a multiple regression analysis to explore the predictive roles of the various variables. A dominance analysis was also used to further investigate the relative importance of each influencing factor. By calculating the mean of the direct, overall, and partial effects of each independent variable, a dominance analysis can decompose the contribution of each independent variable to the total variance of the dependent variables into a percentage in the predicted variance. This way, the analysis makes itself model-independent and free from the impact of different variable combinations, thus showing the relative importance of each independent variable more accurately (26).

We adopted the testing method of one-to-one online inquiry. Initially, a researcher briefs the purpose and significance of the survey to a participant and obtains informed consent. Participants are asked to answer all questions on the questionnaire, independently, and item by item. If a question arises, the researcher responds efficiently via the network. The testing time is 5–10 min. After all questionnaires are completed and collected, some are selected randomly for online one-on-one interviews. Our research was approved by the Ethics Review Committee at the University where the lead researchers work. We used SPSS 22.0 for all statistical analyses.

## Research Results

### Characteristics of Life Meaning and Social Adaptation

Overall, participants scored low on meaning in life, below-average on life goal, and at the medium level on social adjustment (Table 2).

**Table 2:** Descriptive statistics of MLS and SAS scores among undergraduates.

**Variables**	**Gender**		**Left-behind experience**		**Only child**		**Origin**	
	**Male**	**Female**	**Have**	**Have not**	**Yes**	**No**	**Urban**	**Rural**
	**(*n =* 463)**	**(*n =* 525)**	**(*n =* 421)**	**(*n =* 567)**	**(*n =* 436)**	**(*n =* 552)**	**(*n =* 467)**	**(*n =* 521)**
Life enthusiasm	3.47 ± 1.08	3.44 ± 0.98	3.66 ± 0.95	3.30 ± 1.06	3.43 ± 1.08	3.48 ± 1.26	3.36 ± 1.09	3.54 ± 0.96
Life goal	2.99 ± 1.42	2.96 ± 1.21	3.14 ± 1.32	2.85 ± 1.31	3.00 ± 1.38	2.96 ± 1.26	2.87 ± 1.36	3.07 ± 1.27
Life value	3.19 ± 1.29	3.21 ± 1.10	3.33 ± 1.21	3.10 ± 1.17	3.20 ± 1.23	3.20 ± 1.26	3.15 ± 1.22	3.24 ± 1.17
Life freedom	3.21 ± 1.15	3.13 ± 1.08	3.31 ± 1.04	3.06 ± 1.15	3.18 ± 1.14	3.16 ± 1.26	3.06 ± 1.18	3.26 ± 1.05
**Total score on** **Life meaning**	3.22 ± 0.96	3.18 ± 0.90	3.36 ± 0.89	3.08 ± 0.94	3.20 ± 0.97	3.20 ± 0.26	3.11 ± 0.98	3.28 ± 0.88
Satisfaction	3.27 ± 0.88	3.26 ± 0.73	3.14 ± 0.78	3.35 ± 0.81	3.30 ± 0.83	3.24 ± 0.26	3.34 ± 0.80	3.20 ± 0.80
Emotional adaptation	3.24 ± 0.47	3.23 ± 0.45	3.20 ± 0.46	3.26 ± 0.46	3.25 ± 0.45	3.23 ± 0.26	3.26 ± 0.47	3.21 ± 0.45
Study adaptation	3.22 ± 0.65	3.22 ± 0.60	3.15 ± 0.60	3.28 ± 0.63	3.23 ± 0.63	3.22 ± 0.26	3.27 ± 0.63	3.18 ± 0.61
Occupational adaptation	3.29 ± 0.53	3.33 ± 0.48	3.28 ± 0.49	3.33 ± 0.52	3.30 ± 0.54	3.32 ± 0.26	3.35 ± 0.51	3.27 ± 0.50
Self-adaptation	3.34 ± 0.67	3.36 ± 0.62	3.28 ± 0.63	3.40 ± 0.65	3.36 ± 0.66	3.34 ± 0.26	3.40 ± 0.63	3.30 ± 0.65
Interpersonal adaptation	3.31 ± 0.66	3.33 ± 0.64	3.24 ± 0.62	3.38 ± 0.66	3.35 ± 0.66	3.30 ± 0.26	3.38 ± 0.64	3.26 ± 0.65
Campus adaptation	3.32 ± 0.63	3.40 ± 0.59	3.31 ± 0.59	3.40 ± 0.62	3.36 ± 0.63	3.36 ± 0.26	3.40 ± 0.64	3.33 ± 0.58
**Total score on social adaptation**	3.28 ± 0.53	3.31 ± 0.47	3.23 ± 0.47	3.35 ± 0.52	3.31 ± 0.52	3.29 ± 0.26	3.34 ± 0.50	3.25 ± 0.50

A multi-factor ANOVA of two variables suggested that there were main and interaction effects for each dimension of meaning in life and social adjustment (Table 3).

**Table 3:** Multi-factor ANOVA of MLS and SAS scores (*n* = 988).

**Dependent variables**	**Independent variables**	***F***	***df***	***p***	** $\eta_{p}^{\text{2}}$ **
Life enthusiasm	Having left-behind experience or not	26.266	1	0.000	0.026
	Gender * Origin	5.534	1	0.019	0.006
Life goal	Having left-behind experience or not	8.380	1	0.004	0.009
Life value	Having left-behind experience or not	10.144	1	0.001	0.010
	Having left-behind experience or not * Being an only child or not	8.092	1	0.005	0.008
Life freedom	Having left-behind experience or not	12.651	1	0.000	0.013
	Having left-behind experience or not * Origin	5.538	1	0.019	0.006
Total score on life meaning	Having left-behind experience or not	20.552	1	0.000	0.021
	Having left-behind experience or not * Being an only child or not	5.776	1	0.016	0.006
Satisfaction	Having left-behind experience or not	10.048	1	0.002	0.010
Emotional adaptation	Gender * Being an only child or not * Origin	9.600	1	0.002	0.010
Study adaptation	Having left-behind experience or not	6.689	1	0.010	0.007
	Gender * Being an only child or not	4.862	1	0.028	0.005
	Gender * Being an only child or not * Origin	4.236	1	0.040	0.004
	Gender * Having left-behind experience or not * Being an only child or not * Origin	4.168	1	0.041	0.004
Occupational Adaptation	Gender * Being an only child or not * Origin	5.474	1	0.020	0.006
Self-adaptation	Having left-behind experience or not	6.609	1	0.010	0.007
	Gender * Being an only child or not * Origin	5.033	1	0.025	0.005
Interpersonal Adaptation	Having left-behind experience or not	4.325	1	0.038	0.004
Campus Adaptation	Having left-behind experience or not	4.779	1	0.029	0.005
	Gender * Origin	4.614	1	0.032	0.005
Total Score on Social Adaptation	Having left-behind experience or not	8.041	1	0.005	0.008
	Gender * Being an only child or not * Origin	4.342	1	0.037	0.004

#### Characteristics of Life Meaning

Concerning the MLS dimensions on life enthusiasm, the main effect of having a left-behind experience or not was significant (*p* < 0.001), with having a left-behind experience greater than not having a left-behind experience. The interaction effect between gender and origin was also significant (*p* < 0.05); according to simple effect tests, females scored lower than males among urban participants (*p* < 0.05), and urban scored lower than rural among female participants (*p* < 0.001). On life goal, the main effect of having a left-behind experience or not was significant (*p* < 0.01), with having a left-behind experience greater than not having a left-behind experience. On life value, the interaction effect between having a left-behind experience or not and being an only child or not was significant (*p* < 0.01); according to simple effect tests, having a left-behind experience was greater than not having a left-behind experience among only-child participants (*p* < 0.001), and only-child participants was greater than non-only-child participants, among participants with a left-behind experience (*p* < 0.01). On life freedom, the interaction effect between having a left-behind experience or not and origin was significant (*p* < 0.05); according to simple effect tests, not having a left-behind experience was less than having a left-behind experience among urban participants (*p* < 0.001), and urban was less than rural among participants without a left-behind experiences (*p* < 0.01). On the total score on meaning in life, the interaction effect between having a left-behind experience or not and being an only child or not was significant (*p* < 0.05); according to simple effect tests, having a left-behind experience was greater than not having a left-behind experience among only-child participants (*p* < 0.001), and only-child participants were greater than non-only-child participants, among participants with a left-behind experience (*p* < 0.01).

#### Characteristics of Social Adaptation

Concerning the SAS dimensions on satisfaction, the main effect of having a left-behind experience or not was significant (*p* < 0.01), with not having a left-behind experience greater than having a left-behind experience. On emotional adaptation, the interaction effect among gender, being an only child or not, and origin was significant (*p* < 0.01); according to a simple effect test, urban was greater than rural among female participants (*p* < 0.05). On study adaptation, the interaction effect among gender, having a left-behind experience or not, being an only child or not, and origin was significant (*p* < 0.05). According to simple effect tests, not having a left-behind experience was greater than having a left-behind experience among male and female participants under the interaction effect between gender and having a left-behind experience or not (*p* < 0.05); urban was greater than rural among females under the interaction effect between gender and origin (*p* < 0.05); not having a left-behind experience was greater than having a left-behind experience among urban participants under the interaction effect between origin and having a left-behind experience or not (*p* < 0.05); urban was greater than rural among non-only-child participants under the interaction effect between origin and being an only child or not (*p* < 0.05); and not having a left-behind experience was greater than having a left-behind experience among only-child participants under the interaction effect between being an only child or not and having a left-behind experience or not (*p* < 0.05). On occupational adaptation, the interaction effect among gender, being an only child or not, and origin was significant (*p* < 0.05), with urban greater than rural among females (*p* < 0.05) and urban greater than rural among non-only child participants (*p* < 0.05). On self-adaptation, the main effect of having a left-behind experience or not was significant (*p* < 0.05), with not having a left-behind experience greater than having a left-behind experience. The interaction effect among gender, being an only child or not, and origin was significant (*p* < 0.05), with urban greater than rural among females (*p* < 0.01). On interpersonal adaptation, the main effect of having a left-behind experience or not was significant (*p* < 0.05), with not having a left-behind experience greater than having a left-behind experience. On campus adaptation, the main effect of having a left-behind experience or not was significant (*p* < 0.05), with not having a left-behind experience greater than having a left-behind experience. The interaction effect between gender and origin was significant (*p* < 0.05), with females greater than males among urban participants (*p* < 0.01) and urban greater than rural among females (*p* < 0.01). On the total score on social adjustment, the main effect of having a left-behind experience or not was significant (*p* < 0.01), with not having a left-behind experience greater than having a left-behind experience. The interaction effect among gender, being an only child or not, and origin was significant (*p* < 0.05), with urban greater than rural among females (*p* < 0.001) and among non-only-child participants (*p* < 0.05).

### Correlation and Regression Analysis of Life Meaning and Social Adaptation

Through the multi-factor ANOVA, it was found that having a left-behind experience or not was a key influencing factor on meaning in life and social adjustment. To explore the influence of a left-behind experience on these two aspects from each dimension, we divided the participants into two groups for a correlation and regression analysis.

As can be seen from Table 4, there was a significant negative correlation between participants with and without a left-behind experience on all dimensions of meaning in life and social adjustment (*r*_with_ = −0.25 ~ −0.61, mean *p* < 0.01; *r*_without_ = −0.14 ~ −0.70, mean *p* < 0.05).

**Table 4:** Correlation analysis of life meaning and social adaptation.

	**Variables**	**F1**	**F2**	**F3**	**F4**	**F5**	**F6**	**F7**	**F8**	**F9**	**F10**	**F11**	**F12**
With left-behind experience (*n =* 421)	F2	0.46**											
	F3	0.62**	0.63**										
	F4	0.57**	0.44**	0.49**									
	F5	0.74**	0.87**	0.81**	0.71**								
	F6	−0.49**	−0.09	−0.28**	−0.38**	−0.28**							
	F7	−0.57**	−0.52**	−0.56**	−0.60**	−0.61**	0.46**						
	F8	−0.51**	−0.30**	−0.35**	−0.43**	−0.42**	0.60**	0.59**					
	F9	−0.46**	−0.46**	−0.39**	−0.46**	−0.41**	0.36**	0.66**	0.67**				
	F10	−0.54**	−0.35**	−0.44**	−0.51**	−0.51**	0.61**	0.72**	0.66**	0.63**			
	F11	−0.43**	−0.33**	−0.38**	−0.44**	−0.44**	0.50**	0.67**	0.55**	0.63**	0.71**		
	F12	−0.47**	−0.35**	−0.36**	−0.36**	−0.44**	0.50**	0.65**	0.65**	0.57**	0.62**	0.53**	
	F13	−0.60**	−0.39**	−0.25**	−0.54**	−0.55**	0.74**	0.66**	0.84**	0.79**	0.88**	0.81**	0.80**
Without left–behind experience (*n =* 567)	F2	0.59**											
	F3	0.63**	0.67**										
	F4	0.67**	0.47**	0.54**									
	F5	0.82**	0.87**	0.77**	0.75**								
	F6	−0.54**	−0.14**	−0.34**	−0.47**	−0.34**							
	F7	−0.70**	−0.47**	−0.57**	−0.63**	−0.62**	0.58**						
	F8	−0.57**	−0.34**	−0.37**	−0.51**	−0.46**	0.66**	0.66**					
	F9	−0.53**	−0.43**	−0.41**	−0.56**	−0.44**	0.45**	0.69**	0.70**				
	F10	−0.59**	−0.37**	−0.49**	−0.60**	−0.54**	0.73**	0.77**	0.74**	0.66**			
	F11	−0.58**	−0.34**	−0.43**	−0.57**	−0.51**	0.62**	0.76**	0.63**	0.62**	0.79**		
	F12	−0.58**	−0.38**	−0.47**	−0.55**	−0.54**	0.60**	0.74**	0.66**	0.63**	0.71**	0.70**	
	F13	−0.68**	−0.39**	−0.19**	−0.65**	−0.58**	0.80**	0.74**	0.85**	0.78**	0.91**	0.87**	0.85**

Note: * means p < 0.05, ** means p < 0.01. F1=life enthusiasm, F2 = life goal, F3 = life value,

*F4 = life freedom, F5 = life meaning, F6 = satisfaction, F7 = emotional adaptation, F8 = study adaptation, F9 = occupational adaptation, F10=self-adaptation, F11 = interpersonal adaptation, F12 = campus adaptation, and F13=social adjustment*.

Table 5 shows a significant negative predictive effect of life enthusiasm and life freedom on social adjustment among participants with a left-behind experience, and the same for life enthusiasm, life value, and life freedom on social adjustment among participants without a left-behind experience.

**Table 5:** Regression analysis of life meaning on social adaptation.

	**Variables**	**Social adaptation (with left-behind experience,** ***n** **=*** **421)**		**Social adaptation (without left-behind experience,** ***n** **=*** **567)**	
		**Step one**	**Step two**	**Step one**	**Step two**
Step One	Gender	0.033	0.039	0.008	−0.011
	Grade	−0.040	−0.055	−0.100*	0.000
	Being an Only Child or Not	−0.048	0.026	0.007	−0.007
	Origin	0.030	0.050	0.089	0.013
Step Two	F1		−0.363***		−0.436***
	F2		−0.089		0.060
	F3		−0.074		−0.106*
	F4		−0.283***		−0.328***
	Δ*F*	0.629	85.484***	2.467*	161.189***
	*R* ^2^	0.006	0.457	0.017	0.544
	Δ*R*^2^	0.006	0.451	0.170	0.527

*F1 = life enthusiasm, F2 = life goal, F3 = life value, and F4 = life freedom*.

* means p < 0.05, *** means p < 0.001.

### Dominance Analysis of Life Meaning to Social Adaptation

Life meaning was a significant predictor of social adjustment, but the traditional multiple regression method could not accurately determine the relative importance of each life meaning dimension in influencing social adjustment. Therefore, a further investigation was planned through a dominance analysis.

As shown in Table 6, the contribution of life enthusiasm was strongest among the participants with a left-behind experience for predicting social adjustment. Likewise, the contribution of life enthusiasm was the strongest, and life value the weakest, among the participants without a left-behind experience for predicting social adjustment. Life enthusiasm was the dominant factor that influences social adjustment in both groups.

**Table 6:** Relative importance of each life meaning dimension for social adaptation.

**Variables**	**With left-behind experience (*****n** **=*** **421)**			**Without left-behind experience (*****n** **=*** **567)**			
	***R* ^2^**	**X1 life enthusiasm**	**X2 life freedom**	***R* ^2^**	**X1 life enthusiasm**	**X2 life value**	**X3 life freedom**
—	—	0.368	0.315	—	0.474	0.266	0.425
X1 Life enthusiasm	0.368	—	0.068	0.474	—	0.012	0.065
X2 Life value	0.315	0.121	—	0.266	0.220	—	0.197
X3 Life freedom				0.425	0.114	0.038	—
X1X2	0.436	—	—	0.486	—	—	0.056
X1X3				0.539	—	0.003	—
X2X3				0.463	0.079	—	—
X1X2X3				0.542	—	—	—
Decomposition of *R*^2^		0.245	0.192		0.222	0.080	0.186
Percentage in predicted variance		56.19	44.04		40.96	14.76	34.32

## Discussion

### Characteristics of Life Meaning and Social Adaptation

On the whole, undergraduate students in this study scored slightly below average on meaning in life, and toward the middle on social adjustment during the post-epidemic period. There were significant differences between total scores on meaning in life and social adjustment, and also most dimensions (except emotional and occupational adaptation) related to having a left-behind experience or not. On meaning in life, having a left-behind experience was greater than not having a left-behind experience. The opposite occurred for social adjustment.

For meaning in life, studies have shown that undergraduate students with left-behind experiences underperform relative to other groups (16). There are contradictory results, however, suggesting that during a public health emergency, an epidemic, an individual's mental state will change with the external environment, revealing unique manifestations that last for a longer period (27). The interviews in this research uncovered a deeper view of meaning in life in the participants with a unique left-behind experience. Examples of statements during the epidemic's trial of life and death include: “Life is fragile, but tenacious” and “Find out how to live and don't ask why.” Chinese culture refers to an obstacle faced by an individual as “a great test to mind before a great mission invested” to “strengthen a person's resilience and inadequacies” – optimistic explanations of the hardship in life (28). In this sense, if the lack of parental companionship represents an uncontrollable external challenge, then an undergraduate student's stronger sense of meaning in life is the result of an autonomous choice in this environment. In comparison, undergraduates without a left-behind experience during the epidemic have mostly enjoyed parental companionship before college, which leads to less independence (29), a lack of deeper thinking on meaning in life, and lower scores on meaning in life.

Life enthusiasm is an individual's feeling for his or her current life. In this study, urban females scored lower than urban males and rural females. This is because gender and origin suggest different perspectives on life and generate different levels of psychological mindedness (30). Life freedom is the autonomy of an individual's life. In this study, urban undergraduate students without a left-behind experience scored lower than both urban and rural undergraduates with such an experience. Undergraduate students with a left-behind experience behave more independently and rely more on themselves to make decisions (29). Since most rural families have poorer conditions compared with urban ones, rural undergraduates act more maturely.

Life value is an individual's identification with his or her value, and the total score on meaning in life reflects an individual's general meaning in life. In this study, only-child undergraduates with a left-behind experience scored higher than non-only-child undergraduates with such an experience and only-child undergraduates without such an experience. This is because undergraduate students with left-behind experiences are more likely to respond negatively to things (31), which eventually fosters negative feelings. But sometimes, this enables a relatively objective evaluation of their own abilities and identification with their life value. Growing up with family love and more social support (32), only-child undergraduates can explore their life value and interpret meaning in a way that reflects the current situation.

On social adjustment, the existing literature suggests that a left-behind experience can encourage undergraduates to live more positively, leading to better social adjustment, compared to those without such an experience (3). But in this study, in terms of social adjustment, the undergraduate students with a left-behind experience scored lower than those without. On the one hand, this might be relevant to the absence of parental roles during childhood socialization for those with a left-behind experience (19). Then in turn, their social adaptability is lower during the epidemic. On the other hand, most undergraduates and parents are bound together at home during this difficult time. Thus, the major changes in family structures, parent-child patterns, and economic conditions, impact undergraduates with a left-behind experience far more than those who have spent more time with their parents. During this unique time, family relationships can directly influence left-behind children's social adjustment, and therefore, their behavior (33). When interviewed about social adjustment, undergraduate students with a left-behind experience often emphasized the impact of family changes: “I feel like the epidemic has caused more family conflicts.” As this group of students devote more cognitive resources to adjusting to the dramatic changes in their families, there is a sharp decline in resources for social adjustment.

Campus adaptation is an individual's ability to enjoy a smooth college life. Previous studies indicated that urban undergraduate students had better campus adaptability than corresponding rural students (34), irrespective to any gender differences (18). But in this study, urban females outperformed urban males and rural females. This may be due to females' higher self-control than males (35, 36). Emotional adaptation is an individual's ability to control and maintain emotions, occupational adaptation is an individual's decision and preparation for a career goal, and self-adaptation is an individual's awareness and evaluation of his/her ego, as well as the maintenance of positive feelings. As for the total score on social adjustment, it is a holistic assessment of an individual's social adjustment. The current study showed a significant interaction effect among gender, being an only child or not, and origin for the above dimensions. In general, urban females performed better than urban males and rural females. Perhaps females have better emotional perceptions, mental expectations for career choice, and self-knowledge, compared to males (35–39). Also, perhaps urban undergraduates have better emotional control and expression, independence, and adaptation, compared to rural undergraduates (34, 40).

Study adaptation is the mental and behavioral process by which an individual achieves equilibrium with his/her learning environment. In this study, undergraduate students without a left-behind experience outperformed those with such an experience. Also, urban females scored higher than urban males and rural females. This is consistent with research that urban undergraduates adjust better to learning environments and undergraduates without a left-behind experience have higher academic achievements (34, 41). But it is inconsistent with the conclusion that males adjust better to learning environments (42, 43). It is likely that studying online requires more self-discipline, especially when teachers' supervision is minimal during an epidemic. When it comes to self-control and self-regulation, females usually win out (36, 44). Apparently, they also show better adaptability for difficult learning situations.

In short, the characteristics of meaning in life and social adjustment for undergraduates with a left-behind experience are not only a reflection of their past, but also an interpretation of meaning in life and a reflection of their social adjustment during a global crisis.

### Correlation, Regression, and Dominance Analysis of Life Meaning, Social Adaptation, and Left-Behind Experience

In this study, there were significant negative correlations and negative predictive relationships, to varying degrees, for each dimension of meaning in life and social adjustment among undergraduate students with or without a left-behind experience. Based on a hierarchical regression analysis, the social adjustment of undergraduates with a left-behind experience was influenced by life enthusiasm and freedom – two factors of meaning in life. The social adjustment of undergraduates without a left-behind experience was influenced by life enthusiasm, freedom, and value – three factors of meaning in life. Unlike those with a left-behind experience, life value entered the regression equation in this group. Based on a dominance analysis, in terms of the prediction of social adjustment, life enthusiasm contributed most of the explained variance (56.19%), among undergraduates with a left-behind experience. Likewise, life enthusiasm contributed the most (40.96%), and life value the least (14.76%), among those without a left-behind experience. Overall, concerning the influence of meaning in life on social adjustment, the two groups of undergraduate students showed certain commonalities in these two aspects, and demonstrated the uniqueness of the contributing variable “having a left-behind experience or not.”

Previous studies concluded that undergraduates' meaning in life was an effective predictor of their social adjustment (20–22). This is in contrast to the current results. Life meaning is an individual's recognition and pursuit of his/her goals in life, while social adjustment is an individual's positive interaction with the environment. Before the COVID outbreak, undergraduate students lived freely on campus as relatively independent adults. They had much more time to imagine and assume what their life would be like and what meaning they might find. Thus, their sense of meaning in life was mostly positive and enabled them to face joys and sorrows of life with optimism. In turn, this enhances social adaptability.

But since the epidemic, life has been more fragile. Students stay at home, not knowing where life might lead them. Meaning in life has become a riddle, and its pursuit is suspended. A survey conducted during 2019–2020, suggests that 18.5% of Chinese undergraduate students are prone to depression, and 8.4% have a tendency toward anxiety (45). Fear, loneliness, anxiety, and depression have become more prevalent. Without thinking as much about the past or future, undergraduate students are more likely to live in the present and try to cherish every moment (45). In an interview, one student pointed out, “One must live to carry love. Life needs communication, help, and company; it wants sunshine, air, activities, and places for free activities; it asks for food and fun; and it demands feeling and thinking. Life is not a past tense, nor is it a present perfect tense. It is always in the present moment. So, cherish life and cherish the moment.”

The idea of living in the moment and suspending the pursuit of meaning in life enables Chinese undergraduate students to detach themselves from the volatile experience, face the epidemic, and cherish the present with the satisfaction of being alive. The entire society changed rapidly with the onset of COVID-19. People were overwhelmed with the sudden anti-epidemic fight, lock-down, and for students this included the transition to online courses. To adapt to these changes and survive every moment of the present, undergraduate students devoted more cognitive resources to social adjustment, hoping to reach a new balance with the epidemic environment, as opposed to devoting those resources for pursuing meaning in life. This is why their meaning in life and social adjustment show a negative correlation and negative prediction, different from what used to be normal. It is likely that this way of coping will continue in the post-epidemic period of regular prevention and control.

The results of the hierarchical regression and dominance analysis suggested more subtle differences between the undergraduate students with and without a left-behind experience in predicting social adjustment on the dimensions of meaning in life. Overall, both life enthusiasm and freedom played an important role in predicting social adjustment for meaning in life, with enthusiasm proving a dominant influence. But to students without a left-behind experience, life value also matters. Life value refers to an individual's identification with his/her life value. Undergraduates without such experience identify their life value not only from their own perception and experience, but also from the people around them, including their parents. This way, they can feel higher social support (46), obtain a greater sense of safety (47, 48), and find it easier to recognize their meaning in life. The identification with life value will give these undergraduates more courage to face the complex epidemic environment and other challenges.

In sum, the differences in the predicted social adjustment of meaning in life between our two groups of Chinese undergraduate students actually reflect the important role of a left-behind experience in shaping an individual's mindset and perspective.

The current study is limited because it relied solely on explicit, consciously controlled self-reports. Life meaning and social adjustment include both explicit and implicit (less conscious) cognitive processes (49, 50). The results of existing research about explicit and implicit processes are ambiguous. Some studies have demonstrated that explicit and implicit processes are independent of each other, while others indicate explicit and implicit measures assess the same construct (51, 52). The present research will inspire us to conduct future studies on the implicit and explicit natures of both meaning in life and social adaptation of Chinese undergraduates with left-behind experiences.

## Conclusion

Overall, Chinese undergraduate students scored low on meaning in life, below-average on life goal, and at a medium level on social adjustment during the epidemic. Undergraduates with a left-behind experience out-performed those without such an experience in terms of meaning in life, but underperformed in terms of social adjustment. Life enthusiasm and freedom of the students in both groups had a negative predictive effect on social adjustment, with life enthusiasm showing greater influence. Life value in the group without a left-behind experience also made an important impact. The epidemic environment and a left-behind experience were key factors influencing the relationship between meaning in life and social adjustment among Chinese undergraduate students.

## Data Availability Statement

The raw data supporting the conclusions of this article will be made available by the authors, without undue reservation.

## Ethics Statement

The studies involving human participants were reviewed and approved by Chongqing University of Arts and Sciences Institutional Review Board. The patients/participants provided their written informed consent to participate in this study.

## Author Contributions

YG: resources. YG, JL, and JW: writing – review & editing, supervision, and methodology. YG and M-QS: writing – original draft. JL: validation. M-QS and JL: data analysis. YG, M-QS, and C-YL: investigation. YG and JL: project administration. All authors contributed to the article and approved the submitted version.

## Conflict of Interest

The authors declare that the research was conducted in the absence of any commercial or financial relationships that could be construed as a potential conflict of interest.

## Publisher's Note

All claims expressed in this article are solely those of the authors and do not necessarily represent those of their affiliated organizations, or those of the publisher, the editors and the reviewers. Any product that may be evaluated in this article, or claim that may be made by its manufacturer, is not guaranteed or endorsed by the publisher.
